# Mangiferin Enhanced Autophagy via Inhibiting mTORC1 Pathway to Prevent High Glucose-Induced Cardiomyocyte Injury

**DOI:** 10.3389/fphar.2018.00383

**Published:** 2018-04-17

**Authors:** Jun Hou, Dezhi Zheng, Wenjing Xiao, Dandan Li, Jie Ma, Yonghe Hu

**Affiliations:** ^1^Department of Pharmacy, Chengdu Military General Hospital, Chengdu, China; ^2^Department of Cardiovascular Surgery, Jinan Military General Hospital, Jinan, China; ^3^Base for Drug Clinical Trial, Xinqiao Hospital, Army Medical University, Chongqing, China

**Keywords:** mangiferin, high glucose, cardiomyocytes, autophagy, mTORC1, cardiotoxicity

## Abstract

Mangiferin functions as a perfect anti-oxidative compound in the diabetic heart, however, the exact mechanism remains to be elucidated. Here, we show the cardioprotective effect of mangiferin under high glucose-induced cardiotoxic condition mainly contributed to enhanced autophagy via suppressing mTORC1 downstream signal transduction. Primary neonatal rat cardiomyocytes were cultured to detect myocytes injury, autophagy, and related signal transduction under different doses of glucose and mangiferin treatment. High glucose (30 mM) reduced autophagic flux, and increased myocyte apoptosis and death compared with normal glucose (5.5 mM) as determined by variation of autophagy markers LC3-II, p62, parkin, GFP-LC3, or mRFP-LC3 fluorescence puncta, cell viability, cleaved caspase 3, cleaved PARP apoptosis indices, reactive oxygen species (ROS), MAO, and PI death indices. Conversely, mangiferin inhibited hyperglycemia associated oxidative stress by reducing ROS, MAO, cleaved caspase 3, and cleaved PARP generation, reestablishing cell viability, mitochondrial membrane potential, and enhancing autophagic flux, thereby preventing myocytes from high glucose-induced toxicity. Furthermore, cardioprotection with mangiferin was potentially related to the decreased mTOR phosphorylation and suppression of mTORC1 downstream signaling pathway. These data indicated the valuable effects of mangiferin on regulation of cardiac autophagy and pointed to the promising utilization for hyperglycemia control.

## Introduction

As an independent risk factor, hyperglycemia leads to myocardial damage in diabetes (Aronson, 2008; Scherer and Hill, 2016; Tanai and Frantz, 2016). Hyperglycemic cardiotoxicity has been validated in numerous studies (Cai and Kang, 2001; Kobayashi et al., 2012; Xu et al., 2012). A high concentration of glucose induces myocytes death directly, which is due to the excessive formation of reactive oxygen species (ROS; Zhang and Shah, 2014). The key role of high glucose in regulating diabetic myocardial damage has been confirmed by assessing a variety of antioxidants that prevent or delay the development of diabetes and its complications, such as diabetic cardiomyopathy (Hou et al., 2012, 2013; Song et al., 2015).

Autophagy is a dynamic system, which uses double-membrane intracellular vesicles to encircle degraded proteins and organelles and transports them to lysosomes (Mellor et al., 2011; Ha et al., 2015). Autophagy under baseline conditions functions to conserve normal cardiovascular morphology and function (Martinet et al., 2007). Autophagy occurs not only in baseline conditions of the healthy heart but also in diabetic heart, which indicates that autophagy plays a decisive role in preserving cardiac function (De Meyer and Martinet, 2009; Kanamori et al., 2015). Recent studies show the relationship between autophagic changes and diabetic heart damage (Gatica et al., 2015). However, whether this correlation represents a causal relationship has not yet been clarified.

Mangiferin, a major glucoside of xanthone in Rhizome Anemarrhena, has an anti-diabetic cardiomyopathic effect (Muruganandan et al., 2005; Hou et al., 2013). Mangiferin reduces blood glucose and ameliorates blood lipid injuries in diabetic animals, which suggests a possible mechanism for this effect. Our previous works showed mangiferin could mitigate diabetic cardiomyopathic progression via reducing ROS and advanced glycation end-product (AGEs) production to prevent diabetic myocardial fibrosis in diabetic rats (Marambio et al., 2010; Zuo et al., 2011). In addition, the increased activity of damaged mitochondria induces autophagy during oxidative stress, which may participate in the development of diabetic cardiomyopathy. Our preliminary experiments showed that mangiferin could enhance LC3-II protein level in diabetic rat hearts. This raises the question – Does mangiferin decrease high glucose-induced cardiotoxicity via regulating autophagy?

In this report, we exposed neonatal rat ventricular cardiomyocytes to high glucose to observe the effect of mangiferin on alleviating myocyte injury, and autophagy regulation. Our study elucidated that mangiferin protects myocytes against high glucose-induced cardiotoxicity by upregulating autophagy via inhibiting the mTORC1 downstream signaling pathway.

## Materials and Methods

### Reagents

Mangiferin, dihydroethidium (DHE), DMSO, and cytosine 1-β-D-arabinofuranoside were purchased from Sigma-Aldrich (St. Louis, MO, United States). Antibodies against LC3, p62, and anti-GAPDH were purchased from Abcam (Cambridge, MA, United States). Antibodies against AMPK, p-AMPK, ACC, p-ACC (Ser79), mTOR, phospho-mTOR (Ser2448), Akt, phospho-Akt (Ser473), phospho-Akt (Thr308), cleaved caspase 3, PARP, p70 S6 kinase, phospho-p70 S6 kinase (T389), S6 ribosomal protein, phospho-S6, 4EBP1, and phospho-4EBP1 (Thr37/46) were purchased from Cell Signaling Technology (Boston, MA, United States). tf-LC3 plasmid (21074) was purchased from Addgene. CCK-8 Kit, JC-1, and MAO ELISA Kit were purchased from Beyotime (Shanghai, China). 2′,7′-dichlorofluorescein diacetate (DCFH-DA) was purchased from Molecular Probes (New York, NY, United States). Mangiferin was dissolved in DMSO.

### Cell Culture

Primary neonatal rat cardiomyocyte were cultured as described previously (Liu et al., 2015). All procedures were approved by the ethical-scientific committee of the Chengdu Military General Hospital. Myocytes were cultured for 48 h in glucose free DMEM. The 5.5 or 30 mM glucose, then treated with mangiferin for 24 h under the high glucose condition. All media osmolality was constructed equalized to that of 30 mM mannitol.

### Adenovirus Generation

The tandem fluorescent mRFP-GFP-LC3 (tf-LC3) allows one to evaluate the extent of autophagosome and autolysosome formation simultaneously, because LC3 puncta labeled with both GFP and mRFP represent autophagosomes, whereas those labeled with mRFP alone represent autolysosomes (Mizushima et al., 2010). The tf-LC3 plasmid construct was purchased from Addgene and used to generate an adenovirus (Ad-tf-LC3) using the shuttle vector pDC316 and the Admax system. Ad-LacZ was used as the control. Cardiac myocytes were transduced with 15 multiplicities of infection (MOIs) of adenovirus for 24 h.

### Cell Viability Evaluation by CCK-8 Assay

Myocardial cell viability was assessed using a CCK-8 assay. The above-mentioned myocytes were incubated in 96-well plates with 3,000 cells in each well, and this was followed by CCK-8 assay (10 μl/well). The absorbance at 450 nm was measured using a Multiskan microplate reader. The cell viability for 5.5 mM glucose group was set at 100%, while the viability for the other groups was expressed as a percentage of the 5.5 mM glucose group.

### Confocal Microscopy

Cardiomyocytes were infected with AdGFPLC3 or Ad-tf-LC3, and then cultured in DMEM with glucose for 48 h. Myocytes were fixed with 4% paraformaldehyde in PBS for 10 min at room temperature. The slides were imaged with a laser scanning confocal microscope (Leica TCS SP5 II, CA, United States). The confocal pictures were photographed at 600× magnification to illustrate the production of GFP-LC3 and/or RFP-LC3 puncta. The number of GFP and mRFP puncta were calculated as previously described (Zhou et al., 2012).

### Mitochondrial Membrane Potential (Δψm) Assay

Myocytes (2 × 10^5^) were inoculated in 5.5 mM glucose medium supplemented with FBS. At 80% confluence, myocytes were pretreated with different doses of mangiferin and then exposed to 30 mM glucose medium for 24 h. Δψm was detected by JC-1 (Perelman et al., 2012). When Δψm rises, JC-1 aggregates generate red fluorescence in the matrix at excitation/emission wavelengths of 585/590 nm; on the contrary, JC-1 aggregates produce green fluorescence in the monomer form at excitation/emission wavelengths of 514/529 nm.

### ROS Detection

Myocyte ROS generation was detected via DCFH-DA staining (Aranda et al., 2013). After a PBS wash, myocytes were incubated with DCFH-DA at 37°C for 30 min. Fluorescent pictures were captured using an inverted fluorescence microscope (DMi1, Leica, CA, United States) and analyzed by Image-Pro Plus 5.0 software.

### MAO Detection

Myocytes (2 × 10^5^) were inoculated in 5.5 mM glucose containing DMEM supplemented with FBS. At 80% confluence, myocytes were pretreated with different doses of mangiferin and then exposed to 30 mM glucose medium for 24 h. Using PBS (pH 7.2–7.4) to dilute the cell suspension, the cell concentration was about one million per milliliter. Cells were repeatedly frozen and thawed to cause the cell destruction and release of the cell contents. The cell suspension was centrifuged for 20 min (2,000–3,000 rpm) and supernatant was collected and subjected to ELISA.

### PI Staining

Myocyte death was determined by PI (1 mg/ml) staining (Jones and Senft, 1985), which was added directly to the medium. PI can enter the dying or dead cell’s membrane and insert into double-stranded nucleic acids. PI positive myocytes were captured by a fluorescence microscope (DMi1, Leica, CA, United States).

### Western Blot Analysis

Myocyte protein samples were subjected to SDS-PAGE, then transferred to PVDF membranes. The membranes were incubated in blocking buffer for 1 h at room temperature. Membranes were incubated with primary antibodies overnight at 4°C, then membranes were incubated in HRP conjugated secondary antibodies for 1 h at room temperature. Immunoreactive bands were visualized by enhanced chemiluminescence (Deng et al., 2015).

### Statistical Analysis

Data are expressed as mean ± SD. The significance of differences between groups was evaluated using one-way or two-way ANOVA test. Significance was set at *P* < 0.05.

## Results

### High Glucose Suppressed Myocytes Autophagy and Enhanced Myocytes Apoptosis

**FIGURE 1:**
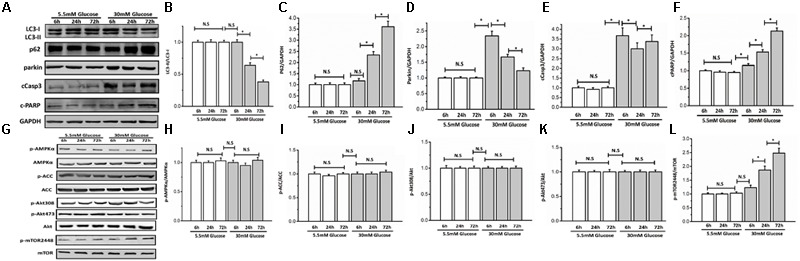
High glucose suppressed autophagy and enhanced myocyte apoptosis. Neonatal rat cardiomyocytes were cultured in DMEM with glucose (5.5 and 30 mM) for 6, 24, and 72 h. Western blots show protein levels of LC3-II, p62, parkin, cCaspase3, cPARP, AMPK, p-AMPKα, ACC, p-ACC, Akt, p-Akt473, p-Akt308, mTOR, and p-mTOR2448. **(A)** Representative image of western blot of LC3-II, p62, parkin, cCaspase3, and cPARP; **(B)** change in LC3-II protein expression; **(C)** change in p62 protein expression; **(D)** change in parkin protein expression; **(E)** change in cCaspase3 protein expression; **(F)** change in cPARP protein expression; **(G)** representative image of western blot of AMPK, p-AMPKα, ACC, p-ACC, Akt, p-Akt473, p-Akt308, mTOR, and p-mTOR2448; **(H)** change in p-AMPKα protein expression; **(I)** change in p-ACC protein expression; **(J)** change in p-Akt308 protein expression; **(K)** change in p-Akt473 protein expression; **(L)** change in p-mTOR2448 protein expression. The quantification of western blots were obtained from at least three independent experiments. Data in the bar graphs were expressed as the mean ± SD, and analyzed by one-way ANOVA. ^∗^*P* < 0.05, significant; N.S., not significant.

To determine whether glucose could influence autophagy, we cultured neonatal rat ventricular myocytes in DMEM with different doses of glucose (5.5 and 30 mM). Autophagy is usually activated quickly in response to stress. Therefore, we performed a time course analysis (6, 24, and 72 h) of autophagy indices, including LC3-II, p62, parkin, AMPK, Akt, and mTOR signaling after high-glucose incubation. The initiation of autophagy is indicated when LC3 is lipidated from LC3-I to LC3-II and incorporated into autophagic vacuoles. SQSTM1/p62, a polyubiquitin binding protein can be degraded by autophagy, and is therefore negatively correlated with autophagy. Parkin, a marker of mitophagy, is recruited to depolarized mitochondria and promotes mitochondrial degradation. Compared with 5.5 mM glucose, high glucose (30 mM) decreased LC3-II and parkin but increased p62 protein expression, which demonstrated that high glucose inhibited myocyte autophagy (**Figures 1A–D**). Autophagy activity is tightly controlled by many positive and negative regulators. The intracellular energy sensor AMPK is a positive regulator of autophagy. However, high glucose did not have a marked effect on AMPK activity as shown by western blot analysis of the phosphorylation of AMPKα at Thr172 and its downstream effector ACC at Ser79, suggesting that AMPK might not participate in autophagy inhibition under high glucose conditions (**Figures 1G–I**). Akt and mTOR are the two classical pathways in autophagy, thus, we determined whether and how Akt and mTORC1 respond to high glucose. Western blot analysis indicated that the Akt pathway was not affected (**Figures 1G,J,K**) at any time tested, while the phosphorylation of mTOR was increased under the high glucose condition (**Figures 1G,L**). To determine whether glucose could influence cardiomyocyte apoptosis, we detected cleaved caspase 3 and cleaved PARP protein levels. As expected, 30 mM glucose led to elevated cleavage of caspase 3 and PARP (**Figures 1A,E,F**).

### Mangiferin Enhanced Autophagy Flux in High Glucose Incubated Cardiomyocytes

**FIGURE 2:**
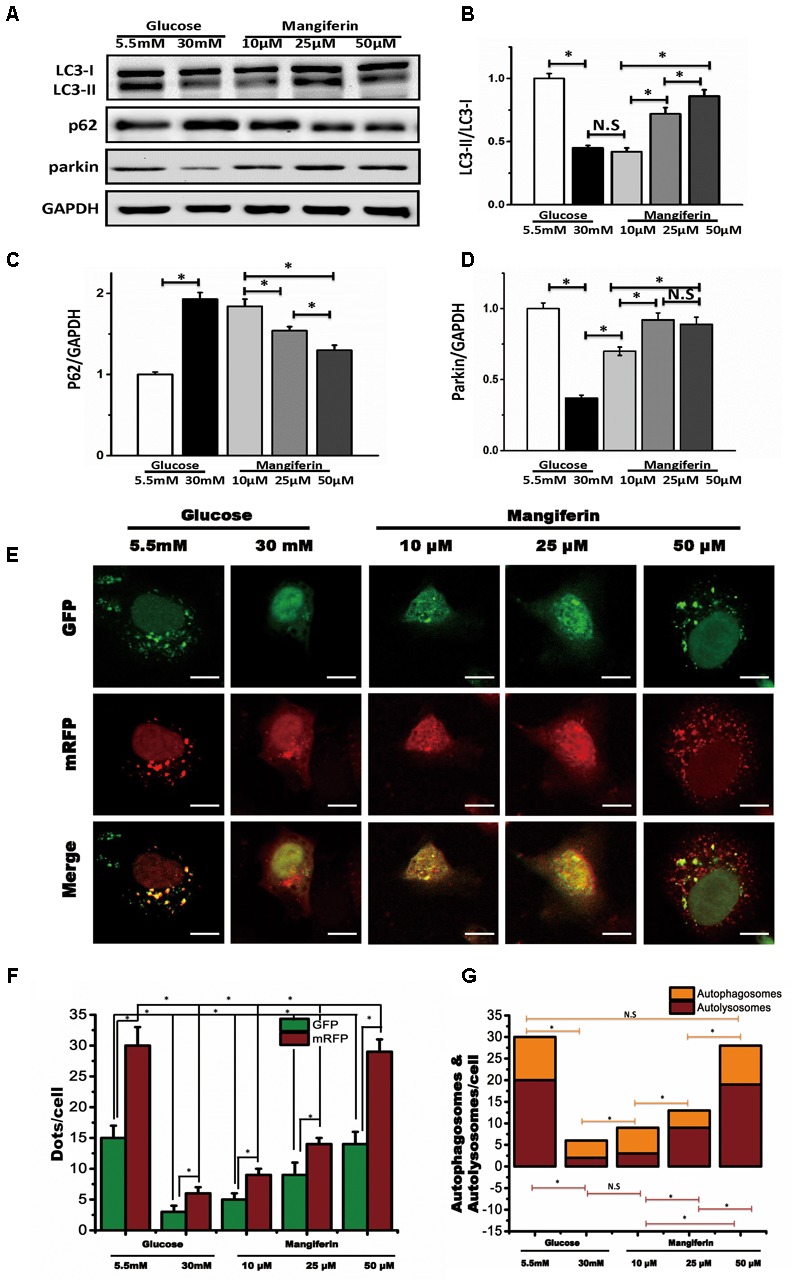
Mangiferin enhanced high glucose-induced inhibited autophagy flux. Western blots show protein levels of LC3-II, p62, and parkin. The quantification of western blots were obtained from at least three independent experiments. **(A)** Representative image of western blot of LC3-II, p62, and parkin; **(B)** change in LC3-II protein expression; **(C)** change in p62 protein expression; **(D)** change in parkin protein expression; **(E)** representative confocal images of fluorescent LC3 punctuates from cardiomyocytes infected with adenovirus expressing tandem fluorescent mRFP-GFP-LC3 (tf-LC3) and cultured with different doses of glucose and mangiferin, showing the formation of autophagosomes and autolysosomes. Autophagosomes were visualized as yellow or orange punctuates in merged images, while red puncta in merged images represent autolysosomes; scale bars represent 20 μm. **(F)** Mean numbers of green and red dots per cell. **(G)** Mean numbers of yellow and red dots per cell. The numbers of fluorescent puncta were counted manually from at least three independent experiments. At least 20 cells were scored in each experiment. Data in the bar graphs were expressed as the mean ± SD, and analyzed by one-way ANOVA. ^∗^*P* < 0.05, significant; N.S., not significant.

According to the above results, we determined optimal experimental duration for the incubation with glucose was 72 h. To determine whether mangiferin could influence autophagy, we cultured neonatal rat ventricular myocyte in DMEM with different doses of mangiferin (10, 25, and 50 μM). With respect to mangiferin treatment, LC3-I to LC3-II conversion and parkin were significantly increased, while expression of p62 was decreased (**Figures 2A–D**). This increase in LC3 net flux and decrease in p62 expression occurred in a dose-dependent manner, suggested that mangiferin enhanced autophagic activity in high glucose cultured myocyte. After observing the autophagic activity change, we next checked the degree of autophagosome and autolysosome formation. Numerous GFP and RFP puncta were seen in myocyte cultured with 5.5 mM glucose, many of which were appeared yellow, indicative of baseline myocyte autophagy (**Figure 2E**). Remarkably, 30 mM glucose not only lowered the number of yellow puncta but also reduced red puncta, revealed the inhibition of the production of autophagosomes and autolysosomes. The number of GFP and mRFP puncta per myocytes were greatly increased after mangiferin treatment in a dose-dependent manner. More red than yellow puncta were visible, showing a significant increase in autolysosome formation in comparison with autophagosomes, and indicated that mangiferin enhanced autophagic flux in a dose-dependent manner. The analysis of GFP and mRFP puncta in myocyte is displayed in **Figures 2F,G**. Taken together, these data implied that high glucose suppressed autophagic flux in myocytes, whereas mangiferin could reverse the reduction of the autophagic flux in a dose-dependent manner.

### Mangiferin Reestablished the Loss of Mitochondrial Membrane Potential (ΔΨm), Suppressed ROS Generation, and Reduced High Glucose-Induced Myocyte Death

**FIGURE 3:**
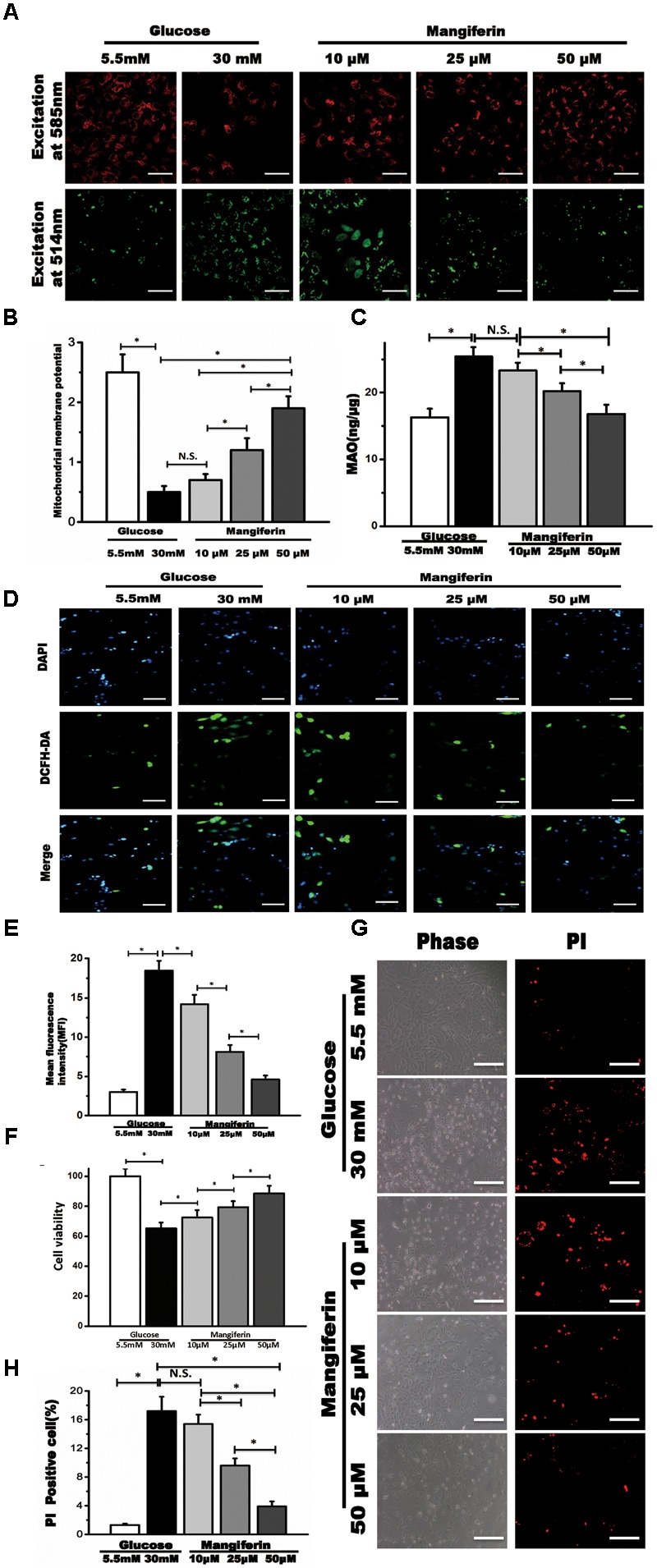
Mangiferin reversed collapse of mitochondrial membrane potential (Δψm) and suppressed ROS generation in high-glucose incubated primary myocytes. **(A)** The Δψm was viewed by JC-1 labeling with fluorescence microscopy, scale bar represents 100 μm; **(B)** quantification of Δψm. The numbers of fluorescent puncta were counted manually from at least three independent experiments. At least 20 cells were scored in each experiment; **(C)** the quantification of MAO; **(D)** the fluorescence microscopic images of ROS detection by DCFH-DA staining in 5.5 mM glucose, 30 mM glucose, and mangiferin treatment (10, 25, and 50 μM) respectively, scale bar represents 50 μm; **(E)** the quantification of intracellular ROS by analyzing mean fluorescent intensity; **(F)** the quantification of cell viability; **(G)** cell death was determined by PI staining, scale bar represents 100 μm; **(H)** the quantification of cell death. Data were expressed as mean ± SD and analyzed by one-way ANOVA (*n* = 4). ^∗^*P* < 0.05, significant; N.S., not significant.

Convincing evidence indicates that mitochondrial dysfunction is critical to diabetic heart damage, hence, we focused on whether mangiferin could influence myocytes ΔΨm when cultured in high glucose via JC-1 staining. JC-1 aggregates in coupled mitochondria exhibiting red fluorescence. Green fluorescence is produced following loss of ΔΨm. Myocytes in high glucose showed weakened red fluorescence and raised green fluorescence, suggesting the collapse of ΔΨm (**Figure 3A**). Mangiferin efficiently reestablished the ΔΨm, as manifested by more red fluorescence followed by less green fluorescence (**Figure 3B**). MAO resides in the outer mitochondrial membrane and serves as a ROS producing sites in mitochondrial. Compared with the 5.5 mM glucose group, mitochondrial MAO was increased in the high glucose group. Mangiferin significantly suppressed MAO levels in a dose-dependent manner (**Figure 3C**). Compared with the 5.5 mM glucose group, the intracellular ROS levels were increased significantly in the high glucose group. Mangiferin significantly suppressed intracellular ROS levels in a dose-dependent manner (**Figures 3D,E**). As shown in **Figure 3F**, myocardial cell viability in the 30 mM glucose group was lower (*P* < 0.05) than that in the 5.5 mM glucose group according to CCK-8 detection. However, myocardial cell viability was significantly increased in the mangiferin group (dose dependent), compared with the 30 mM glucose group (*P* < 0.05). These findings indicate that mangiferin enhances the viability of cardiomyocytes exposed to high glucose. Myocyte death was detected using PI staining, which assesses the number of dying myocytes disregarding the cause of death (**Figure 3G**). High glucose increased PI-positive myocytes (17.2 ± 2.0% in 30 mM glucose vs. 1.3 ± 0.2% in 5.5 mM glucose) as expected. Myocyte death was reduced by mangiferin in a dose-dependent manner as illustrated by the amount of PI-positive myocytes (17.2 ± 2.0% in 30 mM glucose vs. 3.9 ± 0.7% in 30 mM glucose plus 50 μM mangiferin, **Figure 3H**). In summary, these findings revealed a protective role of mangiferin on myocyte death due to high glucose toxicity.

### Mangiferin Activates Autophagy via Suppressing mTOR Pathway

**FIGURE 4:**
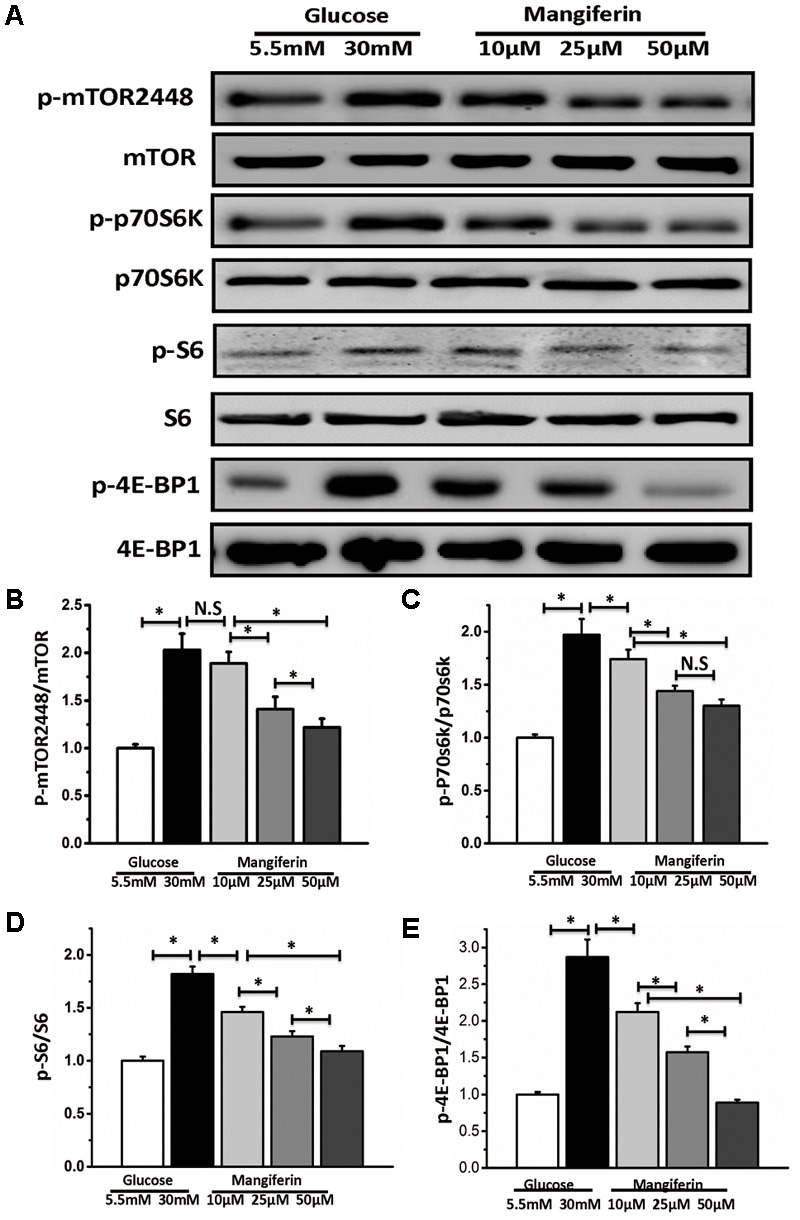
Mangiferin-mediated autophagy activation is dependent on mTORC1 signaling pathway. Western blots show protein levels of mTOR, p-mTOR2448, p70S6K, p-p70S6K, S6, p-S6, 4EBP1, and p-4EBP1. **(A)** Representative images of western blot; **(B)** change in p-mTOR2448 protein expression; **(C)** change in p-p70S6K protein expression; **(D)** change in p-S6 protein expression; **(E)** change in p-4EBP1 protein expression. The quantification of western blots were obtained from at least three independent experiments. Data were expressed as mean ± SD and analyzed by one-way ANOVA. ^∗^*P* < 0.05, significant, N.S., not significant.

Western blot analysis indicated mTORC1 signaling was increased by boosting the phosphorylation of the downstream effectors of mTORC1 under the high glucose condition (**Figures 4A–E**). Thus implying that mTORC1 signaling might respond to autophagy inhibition under high glucose. These results also suggested that mangiferin significantly suppressed the phosphorylation of the mTORC1 pathway, including p-mTOR, p70S6K, p-S6, and p-4EBP1 in a dose-dependent manner (**Figures 4A–E**).

## Discussion

Prior studies have noted the importance of the hyperglycemia association with heart dysfunction in diabetic cardiac damage (Aronson, 2008). However, the cellular and molecular mechanisms leading to cardiotoxicity under hyperglycemic conditions is not clear. In this study, we report that high glucose inhibits autophagic flux in cultured myocytes. Conversely, upregulation of autophagic flux with mangiferin protects myocytes from high glucose-mediated myocyte injury via reducing ROS generation, MAO production, apoptosis, and reestablishment of mitochondrial membrane potential, which contributed to suppressing mTORC1 signal pathway. To the best of our knowledge, this study is the first report of the effect on autophagy of mangiferin in cultured myocytes.

Mitochondrial dysfunction is a critical component of diabetic heart damage (Jung and Lee, 2010). Our previously reported results indicated that the mitochondria within diabetic myocardium underwent morphological changes, such as disruption of cristae, disorganization of sarcomeres, and frequent mitochondrial dense bodies (Hou et al., 2013). We also found a decline in ventricular performance in diabetic cardiomyopathic rats (Hou et al., 2016). In this study, we first sought to determine if myocytes exposed to high glucose, exhibited mitochondrial fragmentation and ROS accumulation, in turn leading to myocyte death. These results were consistent with the data obtained by Yu et al. (2008), and suggested that hyperglycemia induces mitochondrial fragmentation. Mangiferin reestablished the loss of ΔΨm a level observed in normal ventricular performance in rodent models. It is unclear if the decline in ventricular performance occurring in diabetic cardiomyopathy is related to the worsening of myocyte mitochondrial function (Wada and Nakatsuka, 2016). Further research is needed to determine whether the modulation of mitochondrial dynamics can be used as a method to improve mitochondrial function and cardiac performance (Vásquez-Trincado et al., 2016).

Autophagy, a conserved catabolic pathway, occurs in the cytoplasm, degrades organelles (damaged or aged), long-lived proteins, and protein aggregates. High glucose is associated with downregulating the autophagic markers lipidated LC3 and p62 (Marambio et al., 2010). As we know that glucose deprivation could induce autophagy (Moruno et al., 2012), hyperglycemia, a condition of glucose overload, could potentially suppress autophagy (Kobayashi et al., 2012). Myocardial autophagy activation is associated with downregulated production of ROS, fibrosis, and myocyte death. After observing that mangiferin could regulate autophagy, we sought to determine whether the autophagic flux would change. To explore the influence of high glucose on production of autophagosomes and autolysosomes at the same time, we used an adenovirus conveying mRFP-GFP-tfLC3. Thus, the colocalization of GFP and mRFP displayed yellow puncta within autophagosomes, which could be counted by deducting the number of GFP puncta from mRFP puncta. The red mRFP fluorescence that does not colocalize with the green GFP fluorescence signal is indicative of autolysosomes. Mangiferin accelerates cardiac autophagy to prevent high glucose-induced myocyte death by promoting autophagic flux in a dosage-dependent manner.

Both AMPK and mTOR are energy-sensing key elements that link cellular energy catabolism with the autophagic system to result in improved energy efficiency (Qin et al., 2010). AMPK and Akt are two major upstream effectors of mTORC1 and directly participate in the regulation of autophagy. However, western blot assay of the phosphorylation of AMPK results showed that high glucose had little impact on AMPK and Akt activity. How does this discrepancy occur? Maybe, these self-contradictory results displayed different *in vivo* (diabetic cardiomyopathic tissue) and *in vitro* (high glucose cultured myocytes). Our results were identical to Dr. Satoru Kobayashi’s publication (Kobayashi et al., 2012). Most cardiac studies mention that mTOR is a negative regulator of autophagy (Nemchenko et al., 2011). As expected, our results indicated that mangiferin downregulated mTOR expression in high glucose cultured myocytes, proposing that mangiferin may activate autophagy via suppressing the mTOR pathway. mTORC1, a nutrient-sensing kinase, suppresses cellular catabolic pathways such as autophagy by phosphorylating downstream substrates p70S6K and 4EBP1 (Jung et al., 2010; Li et al., 2013; Heras-Sandoval et al., 2014; Zhang et al., 2016). Our data suggested that mangiferin displays potential as a therapeutic drug for decreasing high glucose-induced cardiotoxicity via suppressing mTOR signaling and enhancing autophagy. The most challenging obstacle for using mangiferin therapeutically, is the low water solubility which constricts the successful transfer for drug usage. Insights regarding use of nanotechnology incorporating mangiferin should be considered in future. Recent studies indicated that nanogels are efficient chemical drug carriers for targeted autophagy activators or inhibitors (Zhang et al., 2017). Further research is needed to investigate the potential of nanogels to increase mangiferin bioavailability.

In summary, mangiferin promotes myocytes survival and inhibits glucose-induced cardiotoxicity as expected in a dose-dependent manner, indicating its valuable role in regulating autophagy under hyperglycemia induced oxidative stress. Moreover, autophagy is emerging as an important target in a number of cardiac diseases and when under oxidative stress. This further indicates a potential for the use of mangiferin to regulate autophagy through the inhibition of mTORC1 in the management of diabetic cardiomyopathy.

## Author Contributions

JH and YH participated in research design. JH, DZ, and JM conducted the experiments. DL and WX performed the data analysis. JH, WX, and DZ wrote the manuscript.

## Conflict of Interest Statement

The authors declare that the research was conducted in the absence of any commercial or financial relationships that could be construed as a potential conflict of interest.

## Abbreviations

4EBP1: elongation factor 4E binding protein
ACC: acetyl-CoA carboxylase
AMPK: AMP-activated protein kinase
CCK-8: Cell Counting Kit 8
DMEM: Dulbecco’s modified Eagle’s medium
GFP: green fluorescent protein
LC3: microtubule-associated protein 1 light chain 3
MAO: monoamine oxidase
mRFP: monomeric red fluorescent protein
mTOR: mammalian target of rapamycin
mTORC1: mTOR complex 1
PARP: poly (ADP-ribose) polymerase
p70S6K: p70 ribosomal protein S6 kinase
PI: propidium iodide
SQSTM1/p62: sequestosome 1
tf-LC3: tandem fluorescent mRFP-GFP-LC3
